# Development of an Emulsion Gel Containing Peanut Sprout Oil as a Fat Replacer in Muffins: Physicochemical, Tomographic, and Texture Properties

**DOI:** 10.3390/gels9100783

**Published:** 2023-09-26

**Authors:** Hyunjin Jeong, Chang-Ki Huh, Ho-Kyung Ha, Jungsil Kim, Imkyung Oh

**Affiliations:** 1 Department of Food Science & Technology, Sunchon National University, Suncheon 57922, Republic of Korea; guswls3979@naver.com (H.J.); hck1008@scnu.ac.kr (C.-K.H.); 2 Department of Animal Science and Technology, Sunchon National University, Suncheon 57922, Republic of Korea; hkha@scnu.ac.kr; 3 Department of Bio-Industrial Machinery Engineering, Kyungpook National University, Daegu 41566, Republic of Korea; jungsil.kim@knu.ac.kr

**Keywords:** peanut sprout oil, resveratrol, fat substitute, W/O emulsion gel, emulsifier, bakery products

## Abstract

Peanut sprouts are known to increase their resveratrol content during germination, leading to cultivation in smart farms. Recently, peanut sprout oil extraction and sales have gained traction; however, processed foods utilizing peanut sprout oil have yet to be developed. In this study, water-in-oil (W/O) emulsion gels were structured with water, peanut sprout oil (PSO), sorbitan monostearate (SMS), and candelilla wax (CW) in different ratios, and their potential as shortening substitutes in muffins was evaluated on physicochemical and sensory properties. PSO comprised 67% unsaturated fatty acids and had higher phospholipid (17.97%) and resveratrol (15.95 µg/L) contents and antioxidant activity (71.52%) compared to peanut oil. The PSO emulsion gels were physically structured without changing their chemical compositions. The SMS and CW ratios were found to have a significant influence on the textural properties, solid fat content, rheology, and crystallization of the emulsion gels. The viscoelastic properties of the emulsion gels showed a higher storage modulus than loss modulus and increased with increasing gelator content. Muffins prepared with emulsion gels were characterized by a harder texture and larger pore size, while in the case of muffins mixed with a ratio of 25% SMS and 75% CW, there was no significant difference in overall preference of sensory evaluation compared to shortening muffins. Thus, these findings reveal the potential utility of PSO as a fat substitute and indicate that W/O emulsion gels are suitable for producing muffins without a loss of quality.

## 1. Introduction

Excessive consumption of trans fats, saturated fats, and total fats is associated with a higher risk of chronic metabolic diseases [1]. Replacing saturated fats with sources rich in unsaturated fatty acids will contribute to reducing the concentration of low-density lipoprotein (LDL) and expecting anti-inflammatory effects in vascular cells [2]. Due to health concerns, the utilization of unsaturated fatty acids in processed foods is becoming increasingly important, and is providing an impetus for ongoing technological developments that will promote their more widespread usage. In this regard, oleogelation, a technology that transfers oil from a liquid to solid state without altering its chemical composition, has potential applicability in the development of fat substitutes. Although oleogels have been proven to mimic the functionality of saturated fats, the issue of their high fat content still remains [3]. Regarding this limitation, the production of oil-in-water (O/W) emulsion gels (structured emulsions or oleogel-based emulsion) is considered a promising approach to reduce fat content [4]. Numerous recent studies have reported the development of emulsion gels and the properties of their dispersed and continuous phases. These studies include an investigation of sausages prepared by substituting pork back fat with emulsion gels [5] and the production of a low-fat mayonnaise using emulsion gels [6]. However, research efforts have tended to focus primarily on O/W emulsion gels, whereas water-in-oil (W/O) emulsion gels have received comparatively limited attention [7,8]. W/O emulsion gels are generally stabilized by the presence of a continuous fat crystal network that confers desirable texture, palatability, and functionality [8]. In addition, compared to O/W emulsion gels, W/O emulsion gels with oil as the external phase showed more favorable sensory properties [9]. W/O emulsion gel stabilizers often consist of waxes such as those of rice bran, candelilla, shellac, and sunflower, and their physicochemical properties, shape, and size are determined by their inherent wax composition, solvent type, and processing parameters [10]. However, given that these materials lack surface-active compounds, the use of waxes as the sole emulsifying agents remains challenging, and consequently, identifying appropriate combinations of waxes and emulsifiers is essential for generating stable W/O systems [8]. Candelilla wax (CW) contains n-alkanes that provide structural properties to several infrequently used matrices, forming crystalline networks in the form of microplatelets that coalesce to form a three-dimensional network characteristic of organogels [11]. Sorbitan monostearate (SMS) facilitates the aggregation of molecules, and consequently, the formation of a three-dimensional network that immobilizes organic solvents [12]. In the study by Godoi et al. [12,13], the effects of binary combinations between CW and SMS in organogels were presented. These two agents (CW and SMS) can be predicted to be effectively combinable as structural agents in the manufacturing of W/O emulsion gels.

The agriculture sector is currently in the process of undergoing a transformation driven by new technologies, which holds considerable promise, given that it will enable this primary sector to move to higher levels of farm productivity and profitability [14]. In agriculture, the Internet of Things (IoT) can be applied to multiple practices, including farm monitoring, irrigation, pest control, and harvesting [15], and is accordingly making a substantial contribution to agricultural operations [16]. Particularly during the germination period, which is the most delicate stage of cultivation, it is crucial to meet various conditions such as providing the right levels of water, oxygen, moisture, temperature, and light. Applying IoT technology during germination can increase seed survival rate and create conditions conducive to better seed germination [17]. There has been a marked increase in the cultivation of sprout vegetables in smart farms using IoT, the diversity of which is increasingly expanding to include the sprouts of crop plants such as peanut, radish, and wheat [18,19,20]. Peanut (*Arachis hypogaea*) is an annual herbaceous legume in the Fabaceae family; its nuts are a rich source of nutrients, including carbohydrates, proteins, fibers, fats, phosphorus, resveratrol, and flavonoids [21,22]. Peanut oil mainly comprises the eight major fatty acids: palmitic (C16:0), stearic (C18:0), oleic (C18:1), linoleic (C18:2), arachidic (C20:0), eicosenoic (C20:1), behenic (C22:0), and lignoceric (C24:0) acids, among which oleic acid (monounsaturated fatty acid (MUFA)) and linoleic acid (polysaturated fatty acid (PUFA)) account for a large portion of the oil [23]. Resveratrol (3,4′5-trihydroxylstilbene), one of the inducible phytoalexins, was found as a natural product to have potential health beneficial properties in the prevention of cardiovascular heart diseases, diabetes, neurodegenerative diseases, and certain types of cancer [22,24]. Peanut plants have been identified as among the richest sources of resveratrol, and moreover, it has been found that the sprouts of peanuts contain larger amounts of resveratrol than any other part of the plant [25]. Complex biochemical changes occur following sprouting, and the contents of resveratrol in peanut differ according to the cultivar and the stage of growth stages. However, there have been very few studies that have assessed the utilization of peanut sprout oil in processed foods [26]. Shortening possesses positive functionalities in processed food, including mouthfeel, tenderness, and flavor, as well as contributing to the structure by aeration in the bakery model; however, it is accompanied by health concerns due to the relatively high content of saturated fatty acids (approximately 40%) and the possible presence of trans fatty acids [27]. Therefore, in order to assess the use of plant-based oils rich in antioxidant compounds such as resveratrol, emulsion gels using peanut sprout oil (PSO), candelilla wax (CW), and sorbitan mono stearate (SMS) were prepared and their physicochemical, rheological, and structural characteristics as shortening substitutes in baked products were investigated.

## 2. Results and Discussion

In order to investigate the physicochemical properties of the PSO used in this study, the specific gravity, phospholipid content, and resveratrol content were analyzed. In terms of specific gravity, values of PSO and PO were shown the significant difference of 0.90 ± 0.01 and 0.92 ± 0.01, respectively (Table 1), with the latter value being consistent with the specific gravity values of PO reported by Shad et al. [26] and Gomaa et al. [28]. Nduka et al. [29] have suggested that the significant differences in specific gravity between PSO and PO appear to be attributed to their distinct structural compositions.

Phospholipids play important roles in the structure and function of cell membrane, act as emulsifiers in food production, and have antioxidant activities [30]. Moreover, the phospholipid content of crude oil has been established to be a key factor influencing oil quality, emulsion gel formation stability, and melting point. For this reason, the phospholipid content of PSO was also examined in the present study. The results revealed that the phospholipid content in PSO was three-fold higher than that in PO, which tends to indicate that PSO enhances the storage stability and functionality of oils (or emulsions). Resveratrol, the primary dietary sources of which are known to be grapes and peanuts [31], is a natural stilbene phytoalexin that has been shown to have beneficial effect on human health [21]. These benefits can be attributed to its antioxidant, anti-inflammatory, chemopreventative, cardioprotective, and estrogenic properties, as well as its interactions with signal transduction pathways [32]. In the present study, the resveratrol contents of PSO and PO were measured by the UPLC-MS/MS method and demonstrated in Table 1. The identification of resveratrol was based on comparisons with the spectra of a commercial resveratrol standard. Both PSO and PO were found to be characterized by the same retention time and fragment ion *m*/*z* values of 227, 185, and 143. Peak identification was determined by the mass spectra and *m*/*z* charge fragmentation patterns compared with those of the reference compound and published data [33]. The resveratrol content of PSO (15.98 ± 1.00 µg/L) was approximately 11-fold higher than in PO (1.45 ± 0.9 µg/L), which is consistent with published data on the resveratrol content and nutritional components in peanut sprouts [34]. According to the research by Limmongkon et al. [33], it has been reported that sprouted peanut extract contains not only resveratrol but also numerous phenolic compounds such as hesperidin, coumaric acid, and their respective stilbene derivatives. However, there has not been much research on the beneficial ingredients of sprout peanut oil, and further research is needed to identify these ingredients through composition analysis.

The antioxidant activities of PSO and PO were confirmed by measurement of 2,2-diphenyl-1-picrylhydrazyl (DPPH) radical scavenging activity. As shown in Table 1, the DPPH radical scavenging activity of PSO and PO was 71.52% and 54.87%, respectively. Increases in DPPH scavenging activity were found to be closely associated with increases in the resveratrol contents of peanut kernels following germination. Adhikari et al. [35] reported that the germination of peanuts is associated with the production of bioactive compounds for plant growth. As presented in this study, the significant increases in resveratrol contents, phospholipid contents, and antioxidant activities in response to germination may provide certain health benefits to the consumers of peanut sprout oil.

The fatty acid compositions of PSO and PO are shown in Table 1. Compared with their saturated fatty acid contents (24.75% to 24.17%), both oils have considerably higher proportions of unsaturated fatty acids (75.25% to 75.83%). The primary fatty acid constituents of PSO and PO include oleic, linoleic, palmitic, stearic, and behenic acids, with no significant differences being detected between the two oils with respect to the contents of these fatty acids, which is consistent with the findings reported by Wang et al. [36]. Similarly, Aljuhaimi and Özcan, who compared non-germinated and germinated peanuts, detected comparable amounts of oleic acid and linolenic acid in samples of both oils [37]. Among unsaturated fatty acids, oleic acid (C18:1) and linoleic acid (C18:2) ranged from 37.45 to 38.07% and 35.59 to 35.62%, respectively. The contents of saturated fatty acids, including palmitic (C16:0), stearic (C18:0), and behenic acid (C22:0), were not observed to be significant difficult between PSO and PO. The high oleic acid content in peanut products contributes to numerous positive biological effects, including the supply of health-promoting compounds like sterols, vitamin E, and choline [38]. Furthermore, oleic acid has been shown to have a positive influence on cardiovascular risk factors, such as unfavorable lipid profiles, high blood pressure, and impaired glucose metabolism [39].

**Figure 1:**
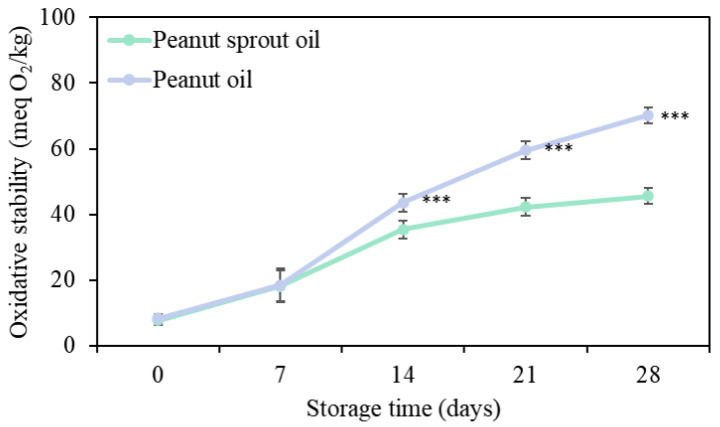
Effect of oils on peroxide value under accelerated storage conditions. An asterisk indicates a significant difference PSO and PO (***: *p* < 0.001).

Emulsion gels are typically prepared by mixing a combination of oil, water, a gelator, and an emulsifier with continuous agitation at high temperature, which may have the adverse effect of promoting the oxidation of oils in emulsion gels. In this regard, peroxide values are used as an index for monitoring oil oxidation and oil quality control during processing and storage [40]. The peroxide values of oils were monitored for a period of 28 days under accelerated conditions (60 °C). Figure 1 shows that the peroxide values of samples can serve as a reliable measure of primary oxidation products in oils. Peroxide values of PSO and PO had a tendency to increase with increasing storage time, and overall, the values obtained for PO were higher than those of obtained PSO. After 14 days of storage time, significant differences were observed between PSO and PO. Particularly, after 28 days of storage, the peroxide values of PO were determined to be higher than 50 meq/kg. Previous studies have highlighted that the oxidative stability of oil is influenced by its antioxidant activity, and oils with low or negligible activity tend to have the highest peroxide values [41,42]. Accordingly, it can be inferred that the resveratrol and phospholipid contents of PSO contribute to the higher resistance of this oil to oxidation, thereby indicating its potential advantages in terms of oxidative stability compared with PO.

**Figure 2:**
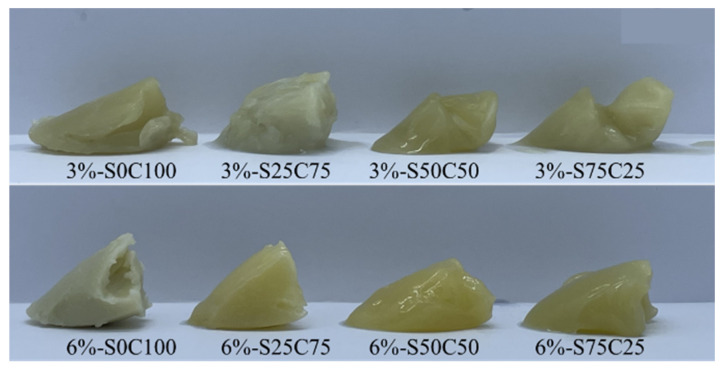
Visual appearances of emulsion gels with different combinations of sorbitan mono stearate and candelilla wax.

Figure 2 exhibits the visual appearance of the emulsion gels prepared from PSO with SMS and CW, which clearly demonstrates that SMS and CW can contribute to the successful conversion of liquid PSO to solid emulsion gels.

**Figure 3:**
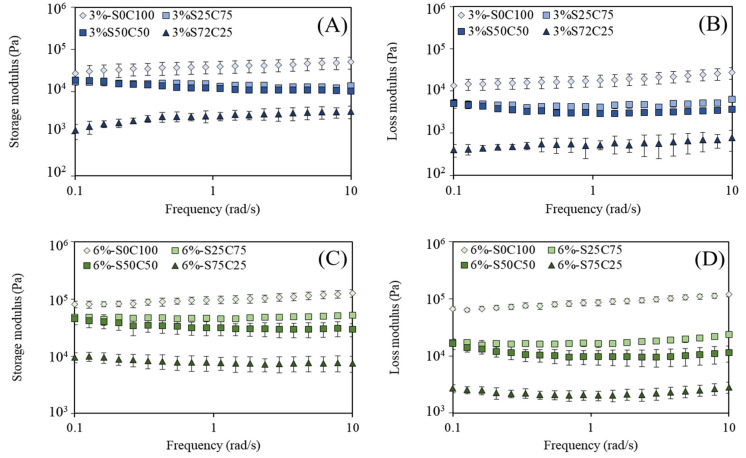
Dynamic viscoelastic properties of 3% and 6% emulsion gels as a function of frequency: 3% emulsion gel storage modulus (**A**); 3% emulsion gel loss modulus (**B**); 6% emulsion gel storage modulus (**C**); and 6% emulsion gel loss modulus (**D**).

The elastic and viscous behaviors of emulsion gels can be assessed based on the storage (G′) and loss (G″) moduli, respectively. Frequency sweep was performed to analyze the viscoelastic properties of emulsion gels. For all the emulsion gel samples, the values obtained for the storage modulus were higher than those of the loss modulus (G’ > G”) throughout the measured frequency amplitudes (Figure 3). These results indicated that the gel-like behavior of emulsion gels predominated over the entire frequency measurement range [43,44]. Emulsion gel containing 6% gelator was found to be characterized by higher elastic modulus values than emulsion gels with 3%. Furthermore, the storage and loss moduli were higher in emulsion gels containing a higher content of CW at the same gelator concentration. These behaviors indicated that flocculated emulsion gels display viscoelastic behavior, and that the emulsion gels also show a tendency for stronger gel behavior with increasing gelator content. Also, the emulsion gel samples remained almost unchanged during the increase in the frequency. This result showed that the emulsion gels had the ability to resist high-frequency oscillation, and that the semisolid structure of emulsion gels was not disrupted by the high-frequency oscillations.

The oil binding capacity (OBC) is one of the key factors in determining whether a new fat formulation will be successfully employed in novel food applications [10]. A higher OBC is indicative of strong forces of attraction, which contribute to restricting phase separation [45]. In the present study, the OBC of emulsion gels increased concomitantly with an increase in the proportion of wax in emulsion gels, thereby highlighting the pronounced effect of wax concentration on this property (Table 2). The highest OBC values were obtained for the 6%-S0C100 sample, which contained the highest CW content, and the OBC declined with an increase in SMS content at the same gelator concentration. This trend was consistent with the rheological data of emulsion gels, implying that the formation of an elastic gel coincided with an increase in OBC. Tavermier et al. [10] also showed that the addition of CW to emulsion gels significantly reduced the percentage of oil loss of the internal fat phase.

The spreadability of emulsion gels was investigated in terms of firmness and work of shear (Table 2). The spreadability of emulsion gel containing vegetable fats is determined by the chemical composition of the type of fat used in their manufacture, as well as the ratio of the aqueous phase to the fat phase and the balance between the liquid and crystalline phase [46]. The values of firmness and work of shear were higher as the gelator concentrations of the emulsion gels increased, and the firmness values within the same gelator concentration were decreased with increasing SMS content.

Differential scanning calorimetry (DSC) can be used to record the heat flow changes caused by phase changes, such as melting, crystallization, and crystal transition in response to changes in temperature, providing data for the analysis of thermal properties [47]. The values of crystallization temperature at the onset of crystallization (T_on_), the peak temperature of crystallization (T_p_), and enthalpy change (ΔH_C_) of emulsion gels were presented in Table 2. Upon cooling, different crystal morphologies may arise, the size and shape of which are dependent on the inherent wax composition, processing parameters (such as cooling and shear regime), and solvent type [8]. The crystallization transitions of emulsion gels occurred at a temperature between 37.28 to 47.92 °C, the peak temperature increased with an increase in gelator content, and the enthalpy increased as the CW content increased. These results are consistent with those obtained for textural analysis, indicating that higher wax contents tend to confer a more stable structure, and are also consistent with the findings of Yılmaz and Öğütcü [48], who similarly reported that the crystallization temperature and enthalpies of organogels increased with increasing wax concentrations.

**Figure 4:**
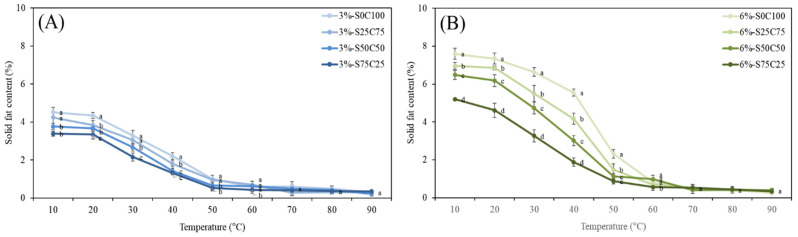
Solid fat content of 3% emulsion gels (**A**) and 6% emulsion gels (**B**). ((**a**–**d**) different letters on the bars indicate that there is a significant difference at *p* < 0.05 according to the temperature).

The solid fat content (SFC) of emulsion gels defines the percentage of solids in fat at certain temperatures, and hence reflects changes in the consistency and plasticity of food products at different temperatures, and is affected by their saturated fatty acid content and polymorphism [49]. The SFC makes an important contribution to determining the quality of baked products, particularly from the perspective of achieving a softer and more uniform structure [50]. Therefore, the SFC of PSO emulsion gels was characterized as a function of temperature. As presented in Figure 4, at a temperature of 10℃, the SFCs of 3% and 6% emulsion gels ranged from 3.38% to 4.52% and 5.20% to 7.60%, respectively. However, at temperatures between 20 to 60 °C, a sharp decline in the SFCs was detected, whereas at temperatures in excess of 60 °C, there was little further change. As well as being predominantly temperature-dependent, the distinct reductions in the SFCs of emulsion gels were observed with increasing levels of SMS. These results suggest that SFC is correlated well with added CW amounts, which is consistent with previous observations that the SFC is mainly determined by the addition of CW to the formulations, whereas the fatty acid composition never changes during the emulsification process [51].

**Figure 5:**
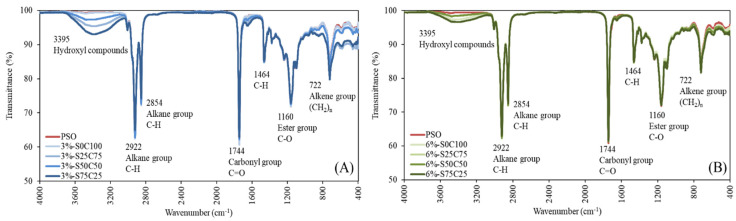
FT-IR spectra of PSO and 3% emulsion gels (**A**) and 6% emulsion gels (**B**) at frequency of 4000–400 cm^−1^.

Fourier transform infrared spectrometry (FT-IR) of PSO and emulsion gels was conducted at various concentrations of gelator in the range of 4000–400 cm^−1^. Figure 5 provides valuable insights into the chemical composition and functional groups of emulsion gels. In the spectra, frequency peaks corresponding to alkene, ester, carboxylic acid, and alkane were observed at 722, 1160, 1464, 1744, 2854, and 2922 cm^−1^ [52,53]. Additionally, the hydroxyl region observed in the emulsion samples at 3395 cm^−1^ is associated with stretching vibrations of OH from water, hydroperoxides, and their breakdown products [54]. This feature is assumed to reflect the generation of hydrogen bonds between the oil and water components introduced during emulsion preparation. It included hydrogen bonding between colloidal particles will help in the construction of the interfacial architecture at the oil–water surface, ultimately forming a stable emulsion [45]. Thus, the spectra of emulsion samples were similar to that of the PSO, except for the hydroxyl group, indicating that PSO was physically solidified without undergoing a change in chemical composition through emulsification.

**Figure 6:**
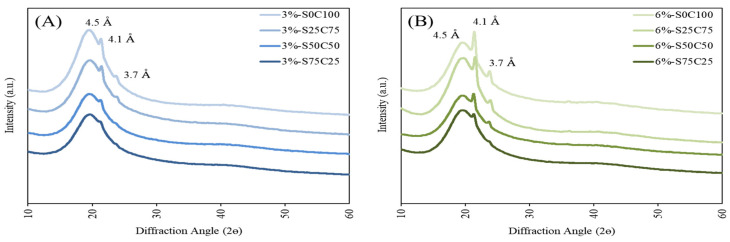
XRD of PSO and 3% emulsion gels (**A**) and 6% emulsion gels (**B**).

The polymorphic nature of crystals in the emulsion gel samples was investigated using X-ray diffraction (XRD) analysis, the resulting diffraction patterns of which are presented in Figure 6. These XRD patterns clearly showed the close similarity of the diffraction peaks of all assessed emulsion gels. All emulsion gels have diffraction peaks at 19.4°, 21.3°, and 23.7°, corresponding to spacings of 4.5, 4.1, and 3.7 Å, respectively, which indicate the presence of β, α, and β′ polymorphic forms in the structure [55]. All the emulsion gels demonstrated a strong peak at 4.1 Å and a weak peak at 3.7 Å, which indicated that the emulsion gels were semisolid systems comprising both crystalline and non-crystalline zones [56]. The diffraction patterns also indicated that the peak intensity increased with an increase in the mass fraction of natural wax, and suggests that the type of gelling agent used has only a minimal effect on the polymorphism of wax-based emulsion gels.

In order to obtain an aerated structure in the final baked product, air cells have to be present into the batter. During mixing, a foam is formed when air is incorporated into the liquid phase. In the presence of CO_2_ and the generated vapor pressure, the air cells undergo expansion, resulting in the formation of final gas cells, which influence the texture of the finished product [57]. Thus, the effects of shortening replacement with emulsion gels on the aeration of the muffin batters were assessed in terms of specific gravity (Table 3). The specific gravity of the emulsion gel muffin batter tended to decrease with higher SMS content, resulting in specific gravities of 1.13, 0.93, 0.88, and 0.85 g/mL for the 6%-S0C100, 6%-S25C75, 6%-S50C50, and 6%-S75C25 batters, respectively. The specific gravity of the control was 0.90, which lies between the values obtained for the 6%-S25C75 and 6%-S50C50 samples.

As clearly shown in Table 3, an increase in the candelilla wax content of emulsion gels resulted in the development of larger air cells and a higher total porosity, as determined by tomographical analysis using a micro-CT. X-ray micro-computed tomography can visualize the inner structure without sample destruction or prior preparation. The ability to measure and visualize the 3D microstructure of food is crucial for understanding characteristics related to processing conditions, such as sensory perception, leading to structural analysis using micro-CT in the field of food as well [58]. Among the assessed samples, the 6%-S0C100 sample was found to have the highest total porosity of 56.48% and a pore size distribution characterized by numerous large pores. In contrast, the shortening sample had a 48.54% total porosity and a uniform muffin crust. These findings tend to highlight the importance of the ratio between candelilla wax and SMS in influencing the specific gravity and porosity of muffins prepared with emulsion gels, which accordingly has potential practical implications with respect to muffin quality and texture.

In general, muffin textural analysis mainly involves texture profiling, using a texture analyzer to determine the hardness, springiness, cohesiveness, and chewiness properties of muffins [59]. As shown in Table 4, there were significant differences in all texture characteristics, and as a result of replacing shortening, the muffin hardness increased and the control sample showed the lowest hardness value. Among the assessed samples, the 6%-S25C75 sample was found to be similar to the control sample in terms of overall textural properties, whereas the highest values of hardness, gumminess, and chewiness were recorded in the 6%-S0C100 sample. These findings are consistent with a previous study demonstrating that the addition of candelilla wax oleogel led to higher hardness levels [60].

The scores of visual appearances, color, flavor, taste, hardness, chewiness, and overall acceptability obtained in sensory analysis of muffins prepared with PSO emulsion gels are shown in Table 5. Although the muffins made with emulsion gels scored significantly lower in visual appearance due to their uneven and lumpy appearance (as shown in Table 3), all samples except 6%-S0C100 did not show a significant difference in flavor and hardness. In the result of overall acceptability, in S25C75 and S50C50 were not observed a significant difference with the control (*p* < 0.05), reflecting favorable texture characteristics. In accordance with Mert and Demirkesen [61], the influence of wax on the sensory aspects of a product result in sensory deterioration in the baked product due to wax odor. However, in this study, when the shortening was replaced with emulsion gel including SMS (S25C75 or S50C50), the flavor improvement effect of the muffin was confirmed. These findings suggest that emulsions can use a shortening replacer, effectively reducing saturated fatty acid content and potentially lowering wax flavor.

The content of resveratrol in muffins was analyzed and is shown in Table 5. The contents of resveratrol of muffins made with emulsion gels were 1.63, 1.55, 1.36, 1.33 µg/g, respectively, and were not detected in muffins using shortening. The higher oil binding capacity or firmness of emulsion gels resulted in the higher resveratrol content of the muffins prepared with emulsion gels. Compared with the resveratrol content in PSO, the resveratrol contents in muffins were detected as approximately 8–15%. These findings suggest that the beneficial resveratrol is well preserved with minimal degradation during the baking process. This result is in agreement with Lyons et al. [62], who noted that heating blueberries for 18 min at 190 °C resulted in the destruction of between 17% and 46% of the resveratrol content. Consequently, muffins made with PSO emulsion gel show promising potential as a nutritional and antioxidant source. Hence, it can be predicted that muffins produced from PSO emulsion gel would have better nutritional quality than muffins produced from shortening.

## 3. Conclusions

Peanut sprout oil was characterized by high resveratrol and phospholipid contents, and antioxidative activity, as well as being rich source of unsaturated fatty acid. The consumption of peanut sprout oil could provide health benefits to consumers. In order to expand the utilization of peanut sprout oil in processed food, in this study, peanut sprout oil was structured using sorbitan monostearate and candelilla wax at different ratios and their thermal, rheological, and structural properties were investigated. Also, their applicability as shortening replacers in muffins was assessed. We found that the ratios of sorbitan monostearate and candelilla wax had a significant influence on the texture, oil-binding capacity, solid fat contents, and viscoelastic properties of these emulsion gels, with water-in-oil emulsion gels containing a higher candelilla wax ratio being characterized by a higher oil binding capacity, solid fat content, firmness, and viscoelasticity. In terms of textural properties, muffins prepared using emulsion gels were found to have higher hardness than those prepared using shortening, although there was no significant difference between the emulsion gels and shortening muffins in the sensory hardness values. In sensory evaluations, the S25C75 muffins showed the highest overall acceptability value among the emulsion gel muffins, and a significant difference was not observed compared to the control, indicating that S25C75 emulsion gel successfully produced muffins without quality loss. Recently, technological advances that facilitate the conversion of liquid vegetable oil to solid-like gel without altering their chemical composition have received increasing attention as an alternative means of replacing solid fat high in saturated fat. Our findings in this will provide useful information for the development of high-quality healthy fat substitutes in the bakery products industry. Accordingly, further extensive studies, for example, on the morphological properties of emulsion gels and oxidative stability of muffins with emulsion gels, should be conducted to promote the use of water-in-oil emulsion gels in food applications.

## 4. Materials and Methods

### 4.1. Chemicals and Reagents

Peanut sprout oil (PSO) was obtained from Ribborn Food & Clinic, Hwaseng, Republic of Korea. This company specializes in the production of PSO extracted at low temperature from peanut sprouts grown in a smart farm. Peanut oil (PO), purchased from a commercial market (Yangpyeong, Republic of Korea), was used as a control sample for comparative purpose. Trans-resveratrol, phosphatidyl choline, and a fatty acid methyl ester (FAME) mixture (no. 18919-1AMP) were purchased from Sigma-Aldrich Co., Ltd. (St. Louis, MO, USA). DPPH (2, 2-diphenyl-1-picrylhydrazyl) was purchased from Alfa Aesar (Tewksbury, MA, USA). And HPLC-grade solvents were purchased from Daejung Chemical & Metals Co. Ltd. (Siheung, Gyeonggi, Republic of Korea). Other reagent-grade chemicals were purchased from Daejung Chemical Co. (Seoul, Republic of Korea).

### 4.2. Characterization of Peanut Sprout Oil

#### 4.2.1. Measurement of Specific Gravity

The specific gravities of PSO and PO were measured using a pycnometer. Empty bottles (*W*_0_) or those filled with oil sample (*W*_1_) and water (*W*_2_) were weighed and the specific gravity was calculated using the following equation:

$$Specific\;gravity=\frac{W_{1}-W_{0}}{W_{2}-W_{0}}$$

#### 4.2.2. Total Phospholipid Determination

The total phospholipid contents of PSO and PO were determined according to a previously described method [63]. A standard calibration curve of six concentrations (0–0.5 mg/mL) was prepared using a commercially obtained pure phosphatidyl choline standard (Sigma-Aldrich, Co.). Briefly, 100 mg of oil was mixed with 2 mL of chloroform and 2 mL of ammonium ferric-thiocyanate (an equal volume of 30 g/L ammonium thiocyanate and 27 g/L ferric acid hexahydrate). The mixture was vortexed for 1 min and centrifuged for 5 min at 1000× *g*. The absorbance was measured at 488 nm using a spectrophotometer (C40: Implen München, Germany).

Standard curve=ax±b

$$Phospholipid\;content=\frac{a*A_{S}}{b}\times 100$$

where *A_s_* is the absorbance of the sample.

#### 4.2.3. Analysis of Resveratrol Content

The contents of trans-resveratrol in oil were determined by ultra-high-performance liquid chromatograph tandem mass spectrometry (LCMS-8060NX, Shimadzu, Japan). Samples of oil (0.5 g) were initially mixed with 1.2 mL of 80% ethanol, and then the mixture was centrifuged at 4000 rpm for 10 min. The collected supernatant was filtered through a 0.22 μm organic membrane filter (Jet Biofil, Guangzhou, China), and the filtrated was analyzed using ZORBAX SB-C18 column (Agilent Technologies, Santa Clara, CA, USA). The mobile phase consisted of solvents A (filtered sterile water containing 0.1% formic acid) and B (HPLC-grade acetonitrile). The flow rate was set to 0.6 mL/min with the follow gradient: 90% A for 3 min, 60% A for 7 min, and 90% A for 11 min. For quantitative analysis, *trans*-resveratrol was used as the standard. In addition, oil extracted from muffins using Soxhlet extraction method was used to determine the amount of resveratrol in muffins. Samples were prepared using a previously described method [64].

#### 4.2.4. Measurement of DPPH Radical Scavenging Activity

The DPPH radical scavenging activities (%) of PSO and PO were conducted according to the method described by Salta et al. [65]. A solution of oil and chloroform (10% *w*/*v*, 6 × 10^−5^ M) was prepared; aliquot (1 mL) was added to a solution of DPPH in chloroform (4 mL). After 30 min, the absorbance was measured at 515 nm using a spectrophotometer (C40: Implen München, Germany). Trolox standards in chloroform (0.001–25 mg/mL) were also measured after the addition of DPPH. The DPPH radical scavenging activity was calculated using the following equation:

$$DPPH\;radical\;scavenging\;activity\left(\%\right)=\frac{\left(A_{c}-A_{t}\right)}{A_{c}}\times 100$$

where *A_c_* is the absorbance of the control and *A_t_* is the absorbance of the sample.

#### 4.2.5. Analysis of fatty acid composition of oils

The fatty acid composition of oils was analyzed using gas chromatography–mass spectrometry (GC-MS, TQ-8040, Shimadzu, Kyoto, Japan). Fatty acid mixture esters (FAME) were separated on a DB-FATWAX UI column (30 m × 0.25 mm × 0.25 μm; Agilent Technologies). The injector operated in the split mode with a 20:1 ratio and at a temperature of 260 °C. The mass detector was set to positive ion electron impact mode at 70 eV with the ion source at a temperature of 300 °C over a scan range of 50–700 *m*/*z*. Helium was used as a carrier gas at a constant flow rate of 0.5 mL/min. Calibration curves were constructed using FAME Mix C8-C24 standards.

#### 4.2.6. Peroxide Value

The peroxide value was determined according to method of Nepote et al. [66]. The oils were subjected to accelerated storage conditions (60 °C) for a duration of 28 days. The oil (1 g) sample was dissolved in 25 mL chloroform: acetic acid (1:2, *v/v*) to which 1 mL of potassium iodide solution was added. The sample was subsequently allowed to react in the dark for 10 min, after which 30 mL of distilled water and 1mL of starch solution (1%) were added, and the sample was titrated with sodium thiosulfate (0.01 N). The peroxide value was expressed as milliequivalents of active oxygen per kg of sample (meqO_2_/kg).

### 4.3. Characterization of Emulsion Gels Containing with PSO

#### 4.3.1. Emulsion Gel Preparation

Sorbitan monostearate (SMS, Ilshinwells Co., Ltd., Seoul, Republic of Korea) and candelilla wax (CW, Kahl GmbH and Co. KG, Trittau, Germany) were purchased from a commercial market. The emulsion gel samples were designated based on the gelator concentrations (3% and 6%) and the ratios of SMS and CW (S0C100, S25C75, S50C50, and S25C75). The emulsion gels (W/O, 20 wt% water) were prepared by melting SMS and CW together with the oil in a water bath at 85 °C for 10 min. Then, the clear oily dispersions were blended with water using a homogenizer (WiseTis-HG-15A, Daihan Co., Wonoju, Republic of Korea) at a speed of 15,000 rpm in an 85 °C water bath for 15 min, followed by cooling and subsequent storage at 5 °C for 3 h [67,68].

#### 4.3.2. Rheological Properties of Emulsion Gels

The rheological properties of emulsion gels were investigated using a controlled stress rheometer (MCR-92, Anton paar, Austria) equipped with a 25 mm parallel plate geometry. The emulsion gels were subjected to oscillation (0.1–10 Hz frequency range) within a linear viscoelastic limit (0.1% strain). The storage moduli (G′) and loss moduli (G″) were measured at a temperature of 5 °C.

#### 4.3.3. Oil Binding Capacity of Emulsion Gels

The oil binding capacity (OBC) of emulsion gels was obtained according to the method described by Abdollahi et al. [69]. The weights of an empty centrifuge tube (A) and one containing 1 g emulsion gel (B) were initially measured. The sample was centrifuged (M13, Hanil Scientific Inc., Gimpo, Republic of Korea) at 12,000× *g* for 15 min. Thereafter, the tube was placed inverted onto filter paper for approximately 10 min to eliminate the detached liquid oil and then re-weighed (C). The oil binding capacity was calculated using the following equation:

$$Released\;oil\left(\%\right)=\frac{\left(B-A\right)-(C-A)}{\left(B-A\right)}\times 100$$

$$il\;binding\;capacity\left(\%\right)=100-Released\;oil(\%)$$

#### 4.3.4. Spreadability of Emulsion Gels

The textural properties of emulsion gels were measured using a texture analyzer TA-XT plus, Stable Micro Systems, Surrey, UK) equipped with a spreadability fixture. Prior to conducting the experiments, the force was calibrated using a 5 kg weight. After filling the sample into a 90 °C conical acrylic mold with a diameter of 45 mm, the sample surface was leveled. The cone probe was positioned in the crosshead of the texture analyzer. The male cone was moved at a test speed of 3 mm/s until it reached 2 mm above the bottom of the female cone. Throughout this process, the maximum force (spreadability) and the work of shear were recorded.

#### 4.3.5. Thermal Properties of Emulsion Gels by Differential Scanning Calorimetry (DSC)

The thermal properties of both the oil and emulsion gels were determined using a differential scanning calorimeter (DSC Q200/TGA Q50, TA Instruments, New castle, DE, USA) according to the method described by Pan et al. [70]. Differential scanning calorimetry (DSC) was characterized by representing the crystallization phase transition. For this analysis, approximately 10 mg samples were sealed in aluminum pans and heated from 25 to 90 °C at the rate of 10 °C/min, and then cooled to −10 °C at the rate of 10 °C/min. TA Instruments Universal Analysis 2000 software was used to plot and analyze the DSC data.

#### 4.3.6. Measurement of Solid Fat Content

The solid fat content of emulsion gel was evaluated using nuclear magnetic resonance (NMR) instrument (MQC+, Oxford Instruments, Oxon, UK). Samples were prepared by filling in the NMR tubes (10 mm diameter) with the gels to about 30 mm high and melting at 90 °C for 30 min. The tubes were placed in an NMR machine and maintained at various measuring temperatures ranging from 10 to 90 °C with intervals of 10 °C for 30 min, followed by the NMR measurement.

#### 4.3.7. Fourier Transform Infrared Spectrometry Analysis

The infrared spectra of oil and emulsion gel samples were obtained using a Fourier transform infrared (FTIR) spectrometer (Spectrum Two, PerkinElmer Inc., Waltham, MA, USA) at room temperature. The scanning range was 4000–400 cm^−1^, and the obtained peaks were analyzed to determine differences among oils and emulsion gels.

#### 4.3.8. X-ray Diffraction Analysis

X-ray diffraction (XRD) analysis of emulsion gels was performed using the X-ray diffractometer (XRD-7000, Shimadzu, Kyoto, Japan). The diffractometer was equipped with Cu Kα source operated at 40 kV and 30 mA in the range of 10–60 °C. The emulsion gels were evenly spread onto glass slide wells at room temperature. The acquired XRD data were analyzed using PCXRD software (version 7.0, Shimadzu, Kyoto, Japan).

### 4.4. Characterization of Muffins

#### 4.4.1. Preparation of Muffins

To evaluate the potential application of emulsion gels to the production of muffins, the shortening typically used in the preparation of muffin was replaced with four different emulsion gels (containing different ratios of SMS and CW). The ingredients used to prepare the muffins were as follows: 200 g soft wheat flour (CJ Co., Seoul, Republic of Korea), 130 g sugar (CJ Co., Seoul, Republic of Korea), 100g shortening (Lotte Co., Seoul, Republic of Korea) or emulsion gel, 16 g non-fat dry milk (Seoul milk Co., Seoul, Republic of Korea), 4 g baking powder (Cheongeun F&B Co., Goyang, Republic of Korea), 1 g salt (CJ Co., Seoul, Republic of Korea), 100 g fresh large eggs, and 100 g water. The muffins were prepared according to the method outlined by Jeong et al. [71]. In this study, the shortening was 100% substituted with emulsion gels containing 6% gelator, which exhibited greater hardness and elastic properties that are reflected in the rheological and texture properties of emulsion gels. The muffins containing emulsion gels were prepared by subtracting the amount of water contained in the emulsion [72]. Each muffin batter (70 g) was placed into muffin pans and baked in a convectional baking oven (SMP-1010, Daeheung Softmeal Co. Ltd. Gwangju, Republic of Korea) at 185 °C for 28 min.

#### 4.4.2. Specific Gravity of Muffin Batter

The specific gravity of muffins was measured by dividing the weight of the batter in a standard container by the weight of an equal volume of water. The specific gravity was calculated with the following equation:

$$Specific\;gravity\left(\frac{g}{mL}\right)=\frac{weight\;of\;muffin\;batter}{weight\;of\;an\;equal\;volume\;of\;water}$$

#### 4.4.3. Analysis of Muffin Textures

The textural properties of muffins were measured using a texture analyzer (TA-XT plus, Stable Micro Systems, Surrey, UK) equipped with a 5 kg load cell and a 50 mm diameter cylinder probe. Cylindrical muffin samples (10 mm in diameter and 30 mm in height) were cut from the center of muffins and compressed twice to a 60% strain at a test speed of 1 mm/s.

#### 4.4.4. Tomographical Analysis

The structural properties of muffins were investigated based X-ray micro-computed tomographic analysis (Skyscan 1174, Bruker, Kontich, Belgium). Cylindrical samples (30 mm diameter × 25 mm height) were cut from the center of muffins and mounted on the scanning platform of the micro-CT. The samples were scanned under the following conditions: 50 kV voltage, 800 μA current, and 0.6 °C angular interval during a 360 °C rotation. Pixel images were reconstructed and analyzed using NRecon (version 1.6.6, Bruker, Konitich, Belgium) and CTAn (Skyscan series, Bruker, Billerica, MA, USA) software.

#### 4.4.5. Sensory Characteristics

Sensory evaluations were performed based on a 9-point hedonic scale ranging from 1 (dislike very much) to 9 (like very much), with attributes in terms of visual appearance, color, flavor, taste, hardness, chewiness, and overall acceptability being assessed. The five types of muffins were assigned random three-digit numbers, and each attribute was independently investigated by a panel of 30 untrained individuals (IRB No. 1040173-202205-HR-008-02) based on their comparability to the control muffins.

### 4.5. Statistical Analysis

All experiments were performed in triplicate with values being expressed as means ± deviation. The data were analyzed statistically using SPSS software (IBM SPSS Statistics 27, IBM Corp., Armonk, NY, USA). The t-test and Duncan’s multiple range test were used for mean comparisons. Differences among the samples were considered significant at a confidence level of 95%.

## Figures and Tables

**Table 1:** Physicochemical properties and fatty acid compositions of PSO and PO.

		PSO	PO	t (*p*)
Specific gravity (g/g)		0.90 ± 0.01	0.92 ± 0.01	−2.036 (0.097)
Phospholipids (%)		17.97 ± 2.25	6.58 ± 1.65	14.072 (0.000) ***
Resveratrol (µg/L)		15.98 ± 1.00	1.45 ± 0.09	25.146 (0.000) ***
DPPH radical scavenging activity (%)		71.52 ± 0.80	54.87 ± 0.35	71.055 (0.000) ***
Fatty acid (%)	Palmitic acid (C16:0)	12.23 ± 0.00	11.97 ± 0.01	−2.173 (0.096)
	Stearic acid (C18:0)	4.38 ± 0.00	4.28 ± 0.00	−2.012 (0.114)
	Oleic acid (C18:1)	37.45 ± 0.01	38.07 ± 0.02	−2.576 (0.062)
	Linoleic acid (C18:2)	35.59 ± 0.01	35.62 ± 0.01	−2.438 (0.093)
	Linolenic acid (C18:3)	0.32 ± 0.00	0.23 ± 0.00	−2.000 (0.116)
	Arachidic acid (C20:0)	2.39 ± 0.00	2.32 ± 0.00	−2.000 (0.116)
	Eicosenoic acid (C20:1)	1.39 ± 0.00	1.49 ± 0.00	−2.828 (0.047) *
	Behenic acid (C22:0)	3.66 ± 0.00	3.60 ± 0.00	−1.871 (0.135)
	Erucic acid (C22:1)	0.49 ± 0.00	0.43 ± 0.00	−0.628 (0.564)
	Lignoceric acid (C24:0)	2.08 ± 0.00	2.00 ± 0.00	−2.828 (0.047) *
	SFA	24.75 ± 0.01	24.17 ± 0.01	−2.127 (0.101)
	USFA	75.25 ± 0.02	75.83 ± 0.03	0.031 (0.977)

An asterisk (*) indicates a significant difference between PSO and PO (*: *p* < 0.05, ***: *p* < 0.001).

**Table 2:** Oil binding capacity and textural and thermal properties of emulsion gels.

Samples	Oil Binding Capacity	Spreadability		Thermal Properties		
		Firmness (*N*)	Work of Shear (*N.s*)	T_on_ (°C)	T_p_ (°C)	ΔH_C_ (J/g)
3%-S0C100	90.23 ± 0.89a	2.09 ± 0.12a	3.15 ± 0.34a	43.28 ± 0.12a	35.64 ± 0.44a	4.40 ± 0.23a
3%-S25C75	76.32 ± 0.55b	0.70 ± 0.70b	1.07 ± 0.25b	40.15 ± 0.09b	32.21 ± 0.33b	3.84 ± 0.65a
3%-S50C50	54.46 ± 2.87c	0.39 ± 0.07c	0.50 ± 0.14c	37.80 ± 0.35c	31.75 ± 1.33b	1.23 ± 0.16b
3%-S75C25	31.62 ± 1.49d	0.09 ± 0.01d	0.13 ± 0.02d	36.16 ± 0.58d	21.24 ± 0.06c	0.48 ± 0.09c
6%-S0C100	97.14 ± 2.05a	6.93 ± 0.44a	12.79 ± 1.54a	47.92 ± 0.06a	41.49 ± 0.60a	8.47 ± 1.76a
6%-S25C75	85.90 ± 1.78b	5.67 ± 1.04b	10.60 ± 1.69b	44.45 ± 0.08b	41.16 ± 1.67a	6.93 ± 1.28a
6%-S50C50	80.58 ± 2.32c	3.74 ± 0.56c	6.49 ± 1.27c	41.88 ± 0.21c	37.95 ± 1.31b	3.83 ± 0.70b
6%-S75C25	49.29 ± 1.53d	0.47 ± 0.03d	0.68 ± 0.05d	37.28 ± 0.39d	21.33 ± 0.05c	3.11 ± 0.43b

((a–d) Means with different letters in the same column differ significantly at *p* < 0.05).

**Table 3:** Specific gravity of muffin batters and tomographical analysis of muffins.

	Control	6%-S0C100	6%-S25C75	6%-S50C50	6%-S75C25
Specific gravity of batters (g/mL)	0.90 ± 0.00c	1.13 ± 0.01a	0.93 ± 0.00b	0.88 ± 0.00d	0.85 ± 0.01e
Micro-CT images	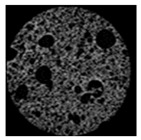	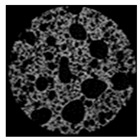	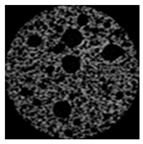	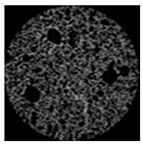	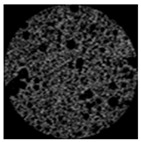
Total porosity (%)	48.54 ± 3.33c	56.48 ± 1.70a	54.46 ± 1.10ab	53.43 ± 0.11b	52.48 ± 1.34b
Open porosity (%)	45.80 ± 3.34b	56.40 ± 1.72a	54.43 ± 1.10a	53.41 ± 0.12a	53.47 ± 2.96a
Closed porosity (%)	0.08 ± 0.01b	0.18 ± 0.04a	0.06 ± 0.04bc	0.04 ± 0.01bc	0.04 ± 0.02c
Pore size distribution (%)	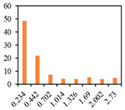	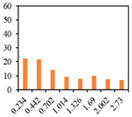	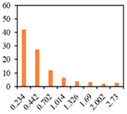	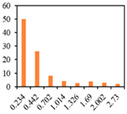	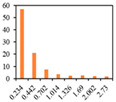
Inner structure	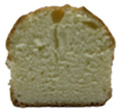	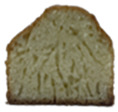	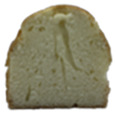	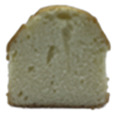	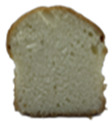

((a–e) Means with different letters in the same row differ significantly at *p*< 0.05).

**Table 4:** Texture analysis of muffins containing shortening and emulsion gels.

	Control	6%-S0C100	6%-S25C75	6%-S50C50	6%-S75C25
Hardness (N)	4.30 ± 0.50b	7.91 ± 0.77a	5.15 ± 0.47b	4.96 ± 0.38b	4.93 ± 0.05b
Springiness (%)	52.24 ± 2.37a	49.99 ± 3.10ab	52.21 ± 0.02a	47.49 ± 2.96b	39.70 ± 2.73c
Gumminess (N)	2.19 ± 0.78b	2.89 ± 0.39a	2.20 ± 0.15c	1.95 ± 0.18d	1.36 ± 0.94d
Chewiness (J)	1.14 ± 0.09b	1.40 ± 0.24a	1.13 ± 0.09c	0.95 ± 0.12d	0.70 ± 0.25e

((a–e) Means with different letters in the same row differ significantly at *p* < 0.05).

**Table 5:** Sensory evaluation and resveratrol content of muffins with emulsion gels.

	Visual Appearance	Color	Flavor	Taste	Hardness	Chewiness	Overall Acceptability	Resveratrol Content of Muffins (µg/g)
Control	7.96 ± 1.08a	7.30 ± 1.14a	7.22 ± 1.38a	7.30 ± 1.52a	6.75 ± 1.07a	7.11 ± 0.58a	7.19 ± 0.83a	N.D.
6%-S0C100	6.36 ± 1.19b	6.93 ± 1.49ab	6.03 ± 1.27b	6.65 ± 1.23ab	6.30 ± 1.18a	6.55 ± 1.28ab	6.48 ± 1.16b	1.63 ± 0.02a
6%-S25C75	6.50 ± 1.10b	6.78 ± 1.34ab	6.76 ± 1.09a	6.18 ± 1.26b	6.77 ± 0.83a	6.76 ± 0.66b	6.74 ± 0.81ab	1.55 ± 0.04b
6%-S50C50	6.59 ± 0.85b	6.72 ± 0.79ab	6.52 ± 0.96ab	7.04 ± 1.33a	6.61 ± 0.99a	6.64 ± 0.91ab	6.62 ± 0.80ab	1.36 ± 0.01c
6%-S75C25	6.78 ± 0.83b	6.26 ± 1.10b	6.71 ± 1.20ab	6.19 ± 1.36b	6.30 ± 0.95a	6.00 ± 0.77c	6.33 ± 0.50b	1.33 ± 0.02d

((a–d) Means with different letters in the same column differ significantly at *p* < 0.05).

## Data Availability

The data presented in this study are available on request from the corresponding author. The data are not publicly available due to the student thesis is into finish process.
